# Population-Based Estimate of Prostate Cancer Risk for Carriers of the *HOXB13* Missense Mutation G84E

**DOI:** 10.1371/journal.pone.0054727

**Published:** 2013-02-15

**Authors:** Robert J. MacInnis, Gianluca Severi, Laura Baglietto, James G. Dowty, Mark A. Jenkins, Melissa C. Southey, John L. Hopper, Graham G. Giles

**Affiliations:** 1 Cancer Epidemiology Centre, Cancer Council Victoria, Victoria, Australia; 2 Centre for Molecular, Environmental, Genetic and Analytic Epidemiology, School of Population Health, The University of Melbourne, Victoria, Australia; 3 Genetic Epidemiology Laboratory, Department of Pathology, The University of Melbourne, Victoria, Australia; IFOM, Fondazione Istituto FIRC di Oncologia Molecolare, Italy

## Abstract

The *HOXB13* missense mutation G84E (rs138213197) is associated with increased risk of prostate cancer, but the current estimate of increased risk has a wide confidence interval (width of 95% confidence interval (CI) >200-fold) so the point estimate of 20-fold increased risk could be misleading. Population-based family studies can be more informative for estimating risks for rare variants, therefore, we screened for mutations in an Australian population-based series of early-onset prostate cancer cases (probands). We found that 19 of 1,384 (1.4%) probands carried the missense mutation, and of these, six (32%) had a family history of prostate cancer. We tested the 22 relatives of carriers diagnosed from 1998 to 2008 for whom we had a DNA sample, and found seven more carriers and one obligate carrier. The age-specific incidence for carriers was estimated to be, on average, 16.4 (95% CI 2.5–107.2) times that for the population over the time frame when the relatives were at risk prior to baseline. We then estimated the age and birth year- specific cumulative risk of prostate cancer (penetrance) for carriers. For example, the penetrance for an unaffected male carrier born in 1950 was 19% (95% CI 5–46%) at age 60 years, 44% (95% CI 18–74%) at age 70 years and 60% (95% CI 30–85%) at age 80 years. Our study has provided a population-based estimate of the average risk of prostate cancer for *HOXB13* missense mutation G84E carriers that can be used to guide clinical practice and research. This study has also shown that the majority of hereditary prostate cancers due to the *HOXB13* missense mutation are ‘sporadic’ in the sense that unselected cases with the missense mutation do not typically report having a family history of prostate cancer.

## Introduction

The *HOXB13* missense mutation G84E (rs138213197) has been reported to be associated with increased risk of prostate cancer [1]. The original study identified 72 case carriers among 5083 tested, but just one control carrier among 1401 tested. Therefore, their point estimate of a 20-fold increased risk was highly imprecise (width of 95% confidence interval (CI) >200-fold) and could be misleading. A more recent paper reported that the mutation was about 8 times more prevalent in cases with a family history of prostate cancer compared with controls; their ratio is also very imprecise as only two controls were carriers [2]. Moreover, one cannot interpret this comparison as a legitimate estimate of increased risk; see [3]. Another recent paper reported a conventional case-control study of 1,525 cases and 1,757 controls that found just two control carriers, and the width of the 95% confidence interval for the odds ratio estimate of 5.8 was 20-fold [4]. That is, estimating risk for rare variants using case-control studies is problematic [5]. Only a newly published study from Sweden had a reasonable number of carrier controls (N = 24 and 37 for the two study populations reported) to estimate risk [6].

For rare variants, a population-based family study can be more informative [5]. To provide accurate and unbiased estimates, it is important that the residual disease correlation within families is taken into account. Failure to adequately adjust for this can lead to upwardly biased penetrance estimates for rare, moderate risk genotypes [7]. The mixed model is one approach that can allow for the simultaneous effect of the *HOXB13* missense mutation G84E and a polygenic component. We have, therefore, used the family cancer histories of a population-based series of early-onset prostate cancer cases (probands), unselected for family history, to estimate from a mixed model the age-specific cumulative risk of prostate cancer (penetrance) for carriers.

## Results and Discussion

Of the 1,505 case probands identified, we successfully genotyped 1,384 (92%). The genotyped and non-genotyped case probands had similar average age at diagnosis (52.4 versus 52.1 years, respectively). We found that 19 (1.4%) case probands carried the *HOXB13* G84E missense mutation. Table 1 shows their pedigree structures at baseline and the clinical details sufficient to estimate penetrance. Six case probands (32%) had a family history of prostate cancer; three had one, and three had two, affected relatives. An additional 22 relatives were genotyped, of whom seven were carriers. Of the nine affected relatives, three were carriers, one was an obligate carrier and the other five could not be genotyped. There were four unaffected carrier relatives (aged 42, 58, 73 and 81 years).

**Table 1:** Clinical details of the 19 *HOXB13* missense mutation G84E carriers (rs138213197), their male relatives and their mothers at baseline, sufficient to calculate the penetrance estimates.*

	Proband's diagnosis details				Male relatives (relationship/observation time/carrier status)	
Family	Age at onset	Gleason's score	Stage	Affected	Unaffected	Carrier status of mother
1	54	7	IIA	None	MU/69/?, S/31/?, S/29/?	?
2	53	6	I	None	F/80/?	?
3	52	7	IIA	None	S/18/?	?
4	53	5	IV	None	B/50/?, B/43/?, B/42/−, B/42/−, B/35/?, F/78/?	+
5	58	7	IIA	None	F/72/?, S/33/?, S/28/?	?
6	52	7	IIA	None	B/50/−, F/76/?	?
7	56	7	IIA	None	B/54/?, S/30/?, S/28/?, PU/79/?	?
8	53	9	III	None	S/25/?, S/21/?	?
9	50	7	IIA	None	F/81/+, S/24/?, MU/80/−, MGF/85/?, PU/78/?, PU/73/+	−
10	52	7	III	None	F/76/?, S/1/?	?
11	54	6	IIA	None	B/49/?, B/43/?, S/30/?	?
12	53	6	III	MU/57/+	F/46/?, S/28/?, HB/42/+, HB/34/−, MGF/65/?	+
13	55	7	IIA	F/68/+, PU/73/?	MU/80/?, PGF/90/?	−
14	47	7	III	F/63/+	MU/70/?, MU/67/?, MGF/83/?, PGF/83/?, S/14/?	−
15	54	9	IV	None	B/50/?, F/64/?, S/18/?	?
16	52	7	IIB	F/63/?, MU/56/?	MU/64/?, MU/36/?	?
17	55	6	IIB	MU/61/?, MU/71/?	B/66/−, B/57/−, S/32/?, S/29/?, S/28/?, F/67/?, MU/67/?, MU/80/?	?
18	55	?	?	None	B/47/−, F/78/?, PU/74/?, S/17/?, S/12/?	?
19	44	7	IIA	F/70/(+)	B/53/+, B/51/−, B/48/?, MU/80/?, MGF/87/?	−

* B, brother; F, father; HB, half-brother; MU, maternal uncle; MGF, maternal grandfather; PU, paternal uncle; PGF, paternal grandfather; S, son.

Observation time began at date of birth and ended at the earliest of the following: date of diagnosis of prostate cancer, date at death, or 31 Dec 2011. Carrier status: + carrier, (+) obligate carrier, − non-carrier, ? unknown carrier status.

**Figure 1:**
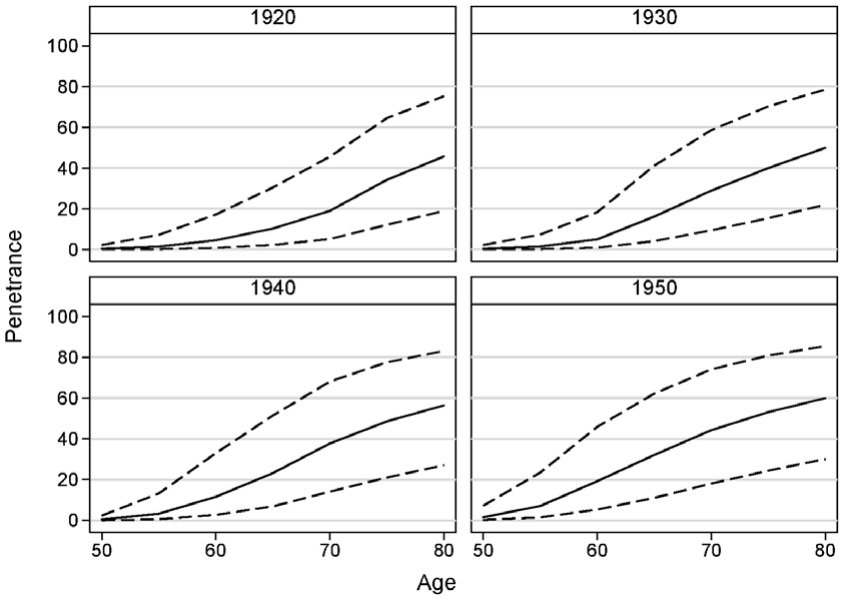
Penetrance (age-specific cumulative risk) (solid line), and 95% confidence limits intervals (dashed lines), of prostate cancer for carriers of the HOXB13 missense mutation G84E (rs138213197) by selected years of birth.

The age-specific incidence for carriers was estimated to be, on average, 16.4 (95% CI 2.5–107.2) times that for the Australian population. There was no evidence that the hazard ratio decreased with age (P = 0.7) or year of birth (P = 0.3), but we had little power to address this issue. The estimated penetrance for carriers, by assumption, varied by year of birth; for example, the estimated penetrance for a male born in 1950 was 19% (95% CI 5–46%) at age 60 years, 44% (95% CI 18–74%) at age 70 years and 60% (95% CI 30–85%) at age 80 years (see Figure 1 and Table 2).

**Table 2:** Penetrance (age-specific cumulative risk) and 95% confidence intervals of prostate cancer for carriers of the *HOXB13* missense mutation G84E (rs138213197) by selected years of birth.

	Birth year							
	1920		1930		1940		1950	
Age	Penetrance	95% CI	Penetrance	95% CI	Penetrance	95% CI	Penetrance	95% CI
50	0.4	(0.1,2.3)	0.4	(0.1,2.3)	0.4	(0.1,2.3)	1.5	(0.2,7.2)
55	1.5	(0.2,7.3)	1.6	(0.2,7.4)	3.2	(0.5,13.2)	7.0	(1.5,23.5)
60	4.6	(0.8,17.2)	5.0	(1.0,18.4)	11.6	(2.7,33.0)	19.2	(5.3,45.9)
65	10.3	(2.3,30.5)	16.2	(4.2,41.2)	23.2	(6.8,51.4)	32.1	(11.0,62.2)
70	19.0	(5.2,45.6)	28.9	(9.5,58.6)	37.7	(14.1,68.1)	44.2	(18.0,74.1)
75	34.2	(12.2,64.5)	40.0	(15.4,70.3)	48.6	(21.0,77.5)	53.1	(24.4,80.9)
80	45.7	(19.0,75.3)	49.9	(22.0,78.6)	56.4	(27.0,83.1)	60.0	(30.1,85.4)

The major strength of our study is that all case probands were sampled from a population-based cancer registry, irrespective of their family history of prostate cancer. Another strength is that we have allowed for residual phenotype correlation within families. Consequently, our estimates of penetrance are unbiased [7].

Our study was focussed on early-onset disease, and sampling restricted to probands diagnosed before the age of 60 years. Therefore, if there are factors that modify risk for carriers, our estimates will be relevant to carriers enriched for those factors. It would be of interest to conduct the same study using probands diagnosed at a later age to see if penetrance is lower. It will also be of interest to see what happens to these families in the future, especially as the proband's brothers move into higher risk age groups. A limitation of this study is the imprecision of the risk estimates (i.e. wide confidence intervals), a consequence of the small numbers of carrier probands and affected relatives, and the limited proportion of relatives with genotype information. Our estimates of age-specific incidence for carriers is consistent with previous studies, [1], [2], [4], [6]. Given we found no evidence that the hazard ratio differed by calendar year, with the caveat of limited power to do so, the varying penetrances depending on year of birth are a consequence of the changing incidence rates over calendar time, most likely due to changes in screening. Up to 50% of our cases would have been unlikely to have come to clinical attention in the absence of PSA testing [8]. Larger family studies of cases with a wider range of age at diagnosis are needed to obtain more precise penetrance estimates. It is also an open question as to whether this penetrance estimate applies to other mutations in the *HOXB13* gene.

Our study has provided an estimate of the cumulative risk of prostate cancer for *HOXB13* missense mutation G84E carriers that can be used to guide clinical practice and research. This study has also shown that, similar to high-risk mutations in other cancer susceptibility genes, the majority of hereditary prostate cancers due to the *HOXB13* missense mutation are ‘sporadic’ in the sense that unselected cases with the missense mutation do not typically report having a family history of prostate cancer [9].

## Materials and Methods

### Ethics statement

The study protocol was approved by the Human Research Ethics Committee of the Cancer Council Victoria. All participants provided written consent to participate in the study.

### Participants

Between 1998 and 2008, all men with histopathologically confirmed carcinoma of the prostate were identified though the population-complete Victorian Cancer Registry. Recruitment included all men younger than 55 years at diagnosis but the upper age limit varied over time according to available numbers, with quotas being filled by randomly sampling additional cases aged from 55 to 59 years. Overall, 68% of eligible men approached participated in the study.

Cases were mailed questionnaires in which they were asked to identify, and provide a detailed cancer history for, their first- and second-degree relatives. Strenuous efforts were made to verify reported prostate cancer diagnoses by use of multiple sources, including cancer reported by relatives, pathology reports, medical records, and death certificates. All probands and selected relatives were asked to provide a blood sample for DNA analysis.

### Genotyping

All probands who donated a blood sample were genotyped for the *HOXB13* missense mutation G84E (rs138213197). For the identified carriers, all relatives from whom a blood sample had been obtained were also genotyped for this missense mutation. Genotyping was performed with the TaqMan assay (Applied Biosystems) using a 384 well format on a LightCycler480, and data were interpreted using LightCycler480 1.5.0 software (Roche Diagnostics, Castle Hill, Australia). The DNA plates contained 31 (1.7%) blind duplicate DNA samples and 6 negative control (blank) wells per 384 well plate that returned high quality genotyping measures (consistent genotyping calls and no evidence of contamination). All identified carriers were confirmed by Sanger sequencing using BigDye v.3.1 chemistry (Applied Biosystems) and chromatograms visualized using chromas (URL:http://www.technelysium.com.au/chromas.html).

### Statistical methods

Prostate cancer incidence *λ_i_*(*t*) for individual *i* at age *t* was assumed to depend on the underlying genotype through a model of the form *λ_i_*(*t*)  =  *λ_0_*(*t*) exp(*G_i_*(*t*)+*P_i_*) where *λ_0_*(*t*) is the age- and birth cohort-specific population incidence at age *t*, *G_i_*(*t*) is the log relative risk corresponding to the *HOXB13* missense mutation G84E carrier status of individual *i* at age *t*, and *P_i_* is the polygenic component that is assumed to be normally distributed with mean 0 and variance *σ_P_* ^2^. The polygenic component is approximated by the hypergeometric polygenic model [10]. *σ_P_* was fixed to equal 2.01 based on a previous segregation analysis [11]. Five-year age-specific population incidences for 1982–2008 in Australia were obtained from the AIHW [12] and smoothed using locally weighted regression techniques [13].

Given probands were sampled independently of family cancer history, for both the penetrance and the HR analyses, we adjusted for ascertainment by conditioning the likelihood for each pedigree on the proband's *HOXB13* missense mutation G84E carrier status, prostate cancer status, and age at diagnosis. We included all first- and second-degree relatives who were ascertained, irrespective of affected status.

Pedigree analyses were performed with the program MENDEL [14]; all other calculations were performed in Stata 11.

## References

[1] Ewing CM, Ray AM, Lange EM, Zuhlke KA, Robbins CM, et al (2012) Germline mutations in HOXB13 and prostate-cancer risk. N Engl J Med 366: 141–149. 22236224 10.1056/NEJMoa1110000 PMC3779870

[2] Breyer JP, Avritt TG, McReynolds KM, Dupont WD, Smith JR (2012) Confirmation of the HOXB13 G84E Germline Mutation in Familial Prostate Cancer. Cancer Epidemiol Biomarkers Prev. 10.1158/1055-9965.EPI-12-0495 PMC3415588 22714738

[3] Byrnes GB, Southey MC, Hopper JL (2008) Are the so-called low penetrance breast cancer genes, ATM, BRIP1, PALB2 and CHEK2, high risk for women with strong family histories? Breast Cancer Res 10: 208. 18557994 10.1186/bcr2099 PMC2481495

[4] Akbari MR, Trachtenberg J, Lee J, Tam S, Bristow R, et al.. (2012) Association Between Germline HOXB13 G84E Mutation and Risk of Prostate Cancer. J Natl Cancer Inst. 10.1093/jnci/djs288 22781434

[5] Hopper JL, Bishop DT, Easton DF (2005) Population-based family studies in genetic epidemiology. Lancet 366: 1397–1406. 16226618 10.1016/S0140-6736(05)67570-8

[6] Karlsson R, Aly M, Clements M, Zheng L, Adolfsson J, et al.. (2012) A Population-based Assessment of Germline HOXB13 G84E Mutation and Prostate Cancer Risk. Eur Urol. 10.1016/j.eururo.2012.07.027 22841674

[7] Gong G, Hannon N, Whittemore AS (2010) Estimating gene penetrance from family data. Genet Epidemiol 34: 373–381. 20397150 10.1002/gepi.20493 PMC3003663

[8] Etzioni R, Cha R, Feuer EJ, Davidov O (1998) Asymptomatic incidence and duration of prostate cancer. Am J Epidemiol 148: 775–785. 9786232 10.1093/oxfordjournals.aje.a009698

[9] Cui J, Hopper JL (2000) Why are the majority of hereditary cases of early-onset breast cancer sporadic? A simulation study. Cancer Epidemiol Biomarkers Prev 9: 805–812. 10952097

[10] Lange K (1997) An approximate model of polygenic inheritance. Genetics 147: 1423–1430. 9383082 10.1093/genetics/147.3.1423 PMC1208263

[11] MacInnis RJ, Antoniou AC, Eeles RA, Severi G, Guy M, et al (2010) Prostate cancer segregation analyses using 4390 families from UK and Australian population-based studies. Genet Epidemiol 34: 42–50. 19492347 10.1002/gepi.20433

[12] AIHW (Australian Institute of Health and Welfare) (2011) ACIM (Australian Cancer Incidence and Mortality) Books. Canberra: AIHW.

[13] Royston P (1991) Lowess smoothing. Stata Tech Bull 3: 7–9.

[14] Lange K, Weeks D, Boehnke M (1988) Programs for Pedigree Analysis: MENDEL, FISHER, and dGENE. Genet Epidemiol 5: 471–472. 3061869 10.1002/gepi.1370050611

